# Speech and Language Therapists’ Perspectives of Virtual Reality as a Clinical Tool for Autism: Cross-Sectional Survey

**DOI:** 10.2196/63235

**Published:** 2025-02-27

**Authors:** Jodie Mills, Orla Duffy

**Affiliations:** 1 Speech and Language Therapy, School of Health Sciences, University of Ulster, 2-24 York Street, Coleraine, BT15 IAP, United Kingdom, 44 2895365308

**Keywords:** virtual reality, VR, autistic, ASD, speech, language, autism, speech and language therapy, speech-language pathology, SLT, immersive, voice, vocal, cross sectional, surveys, questionnaires, experiences, attitudes, opinions, perceptions, perspectives, autism spectrum disorder

## Abstract

**Background:**

Persistent difficulties with social skills form part of the diagnostic criteria for autism and in the past have required speech and language therapy (SLT) management. However, many speech and language therapists are moving toward neuro-affirmative practices, meaning that social skills approaches are now becoming redundant. Research demonstrates that virtual reality (VR) interventions have shown promise in overcoming challenges and promoting skill generalization for autistic children; however, the majority of these focus on social skills interventions. While VR is emerging as an SLT intervention, its application for autism remains unexamined in clinical practice.

**Objective:**

This research aimed to examine speech and language therapists’ knowledge and attitudes toward immersive VR as a clinical tool for autistic children and explore the reasons for its limited integration into clinical practice.

**Methods:**

A web-based cross-sectional survey was available from April 3, 2023 to June 30, 2023. The survey, consisting of 23 questions, focused on VR knowledge, attitudes, and the support required by speech and language therapists to incorporate VR into clinical practice. Dissemination occurred through the Royal College of Speech and Language Therapists Clinical Excellence Networks to recruit speech therapists specializing in autism.

**Results:**

Analysis included a total of 53 responses from the cross-sectional survey. Approximately 92% (n=49) of speech and language therapists were aware of VR but had not used it, and 1.82% (n=1) had used VR with autistic children. Three key themes that emerged were (1) mixed general knowledge of VR, which was poor in relation to applications for autism; (2) positive and negative attitudes toward VR, with uncertainty about autism specific considerations for VR; and (3) barriers to adoption were noted and speech and language therapists required an improved neuro-affirming evidence base, guidelines, and training to adopt VR into clinical practice.

**Conclusions:**

While some speech and language therapists perceive VR as a promising intervention tool for autistic children, various barriers must be addressed before its full integration into the clinical toolkit. This study establishes a foundation for future co-design, development, and implementation of VR applications as clinical tools for autistic children.

This study is the first to explore clinical implementation factors for the use of VR in SLT field, specifically with autistic children. Poor autism-specific VR knowledge, and mixed attitudes toward VR, highlight that specific barriers must be addressed before the technology can successfully integrate into the SLT clinical toolkit.

Speech and language therapists require support from employers, funding, a robust neuro-affirming evidence base, and education and training to adopt VR into practice. Recommendations for a SLT VR education and training program for use with autistic children, are provided.

## Introduction

Virtual reality (VR) uses technology to digitally replicate real world scenarios [1] by providing artificial tactile, visual, and auditory feedback. This allows users to experience environments as if they were real. A VR system connects the user to the virtual environment through sensory modalities, providing sight, sound, and tactile feedback. This can be achieved through different technologies, such as Computer Automated Virtual Environments (CAVE) and immersive head mounted displays (HMDs). These facilitate different levels of interaction, presence, and immersion, such as the perception of being in the real world. In the context of this paper, VR refers to HMDs and CAVE, with the idea of integrating HMDs into speech and language therapists’ clinical toolkits.

Research has demonstrated that VR capitalizes on visual skills, known to be a strength of autistic children, and can facilitate safe, controlled, and customizable practice environments, without negative consequences [2]. This has led to further research suggesting VR-based interventions may be effective for improving socials skills and generalization, enhancing emotional recognition, reducing anxiety, and facilitating sensory integration [3,4]. Indeed, research has demonstrated a moderate evidence base for VR with the autistic population [5]. This is evidenced by a pre-post study by George and Shakul [6], which demonstrated that a VR reality intervention for young autistic adults effectively improved their social and communication skills, and this group preferred the VR intervention in comparison with traditional methods. Furthermore, the study provides evidence that VR-based interventions can be used to complement traditional therapies for autistic service users and suggest future studies should investigate the benefits of combining VR-interventions with speech and language therapy (SLT). While VR has the potential to host communication or language-based interventions for populations such as developmental language disorder, the need to develop neuro-affirming tools to support autistic children is pressing.

As suggested above, a critical lens must be applied to previous VR research. A large proportion of studies with autistic children target social skills training, which is a neuro-affirmative shortcoming. These studies are criticized for using deficit-based interventions that aim to reduce and normalize autistic social behaviors, potentially increasing the risk of masking, negative mental health outcomes, and in some cases, trauma [7]. A growing number of professionals specializing in communication and social skills, such as speech and language therapists, are moving toward neuro-affirming practice [8]. Therefore, this paper does not use ableist language, such as autism spectrum disorder, instead adopting the term autism throughout. This reflects the authors nonpathologizing approach to autism. Neuro-affirming practice is described as support for autistic identity through strength-based, environment first approaches that promote adaptions [9]. They seek to remove environmental and societal barriers, imposed by deficit-based neurotypical standards and facilitate autistic self-advocacy [10]. Despite this, it can be difficult to know how to use resources to meet goals for autistic children. As neurotypical resources may not capitalize on autistic strengths, alternative tools, such as virtual reality (VR), should be explored to understand their application to clinical practice for autistic children [11]. As clinicians involved in the care of autistic children relating to communication and socialization, speech and language therapists’ perspectives may provide insight into clinical applications of VR. Potential neuro-affirming uses have been demonstrated across literature. For example, autistic users have reported VR could help them with (1) anxiety surrounding unknown experiences; (2) concrete, literal thinking; (3) improving self-esteem and positive self-identity; (4) repetitive practice in real-life environments; and (5) making friends [11-14]. However, particularities of autism, such as sensory changes, disassociation, and indeed individualized presentations, may pose a barrier to the use of VR. Given that, VR has not been widely adopted by speech and language therapists in clinical practice, this potential remains unharnessed.

In total, 3 key papers have established a preliminary evidence base for VR as an intervention for autistic children in the SLT field. First, literature review by Bryant et al [15] provided initial support for the use of immersive VR for “communication disability” and autism, in SLT, and indicated there is some potential for every day, functional communication skills developed in VR worlds to transfer into real-world interactions. However, they suggested that technological complexities may act as a barrier to individuals with comorbid intellectual disabilities, and that VR may create ethical and safety issues that should be considered carefully. Second, systematic review by Bailey et al [16] suggested that VR could support several social communication goals for autistic children and those with an intellectual disability. However, results varied greatly, included research with low quality methodology, and outcomes were inconsistent across VR technologies. Therefore, it was difficult to compare the findings. Finally, a study by Ghanouni et al [17] examined the effectiveness of 75 speech and language therapists’ social stories focused on perspective taking. While this indicates VR may be an effective medium for 'intervention' with autistic children, it is difficult to know if research is based on neuro-affirming principles. Evidence underpinning effective and affirming VR interventions in autism requires further development. Overall, Bryant et al [15] shows that VR implementation in SLT practice is still in the early stages. Therefore it is difficult to determine “which [VR] technologies work for whom, in which contexts,” how VR supports SLT tasks and objectives, and the support required [18].

The precursor to using VR in clinical practice is exploring speech and language therapists’ perceptions of VR and appetite for future usage. This may help to bridge the gap between technology and practice. Studies documenting clinician perceptions of VR in adult rehabilitation are positive, but knowledge and attitudes toward VR act as barriers to adoption [19]. For example, perceptions of a lack of SLT evidence, the need for training and guidelines, workplace limitations and side effects are challenging [15,20]. It is necessary to engage SLTs as one of the end users of technology, to reduce discrepancies between their needs and VR capabilities [21]. Ultimately, this may demonstrate if VR is an acceptable tool for autistic children and SLT practitioners. Despite this, there is a gap in literature examining SLT knowledge and attitudes toward VR as a tool for autistic children. Therefore, this should be explored to understand why VR has not yet been adopted into SLT practice.

In this work, we explore speech and language therapists’ knowledge and attitudes toward the use of VR for autistic children. We focused on investigating knowledge and attitudinal factors that contribute to the adoption of technology, and their specific applications to an autistic population. In addition, we explored practical steps to enhance future adoption of VR by SLTs. We sought to answer 3 questions with SLTs practicing in the autism field. First, what knowledge do speech and language therapists have about VR? Second, what are speech and language therapists’ attitudes toward using VR as an intervention for autistic children? Third, what do SLTs need to support the implementation of VR in clinical practice? With this research, we aim to create a basis for future research, co-design, and implementation of VR for autistic children in an SLT setting.

## Methods

### Overview

A web-based questionnaire, reported in accordance with the CHERRIES (Checklist for Reporting Results of Internet E-Surveys) checklist [22], was created to gather qualitative and quantitative data exploring speech and language therapists’ knowledge and attitudes toward VR as a tool for autism. This was a cross-sectional study, with data representing a snapshot of speech and language therapists’ perspectives at a single point in time.

### Participant Recruitment

Recruitment used convenience sampling, and participants were eligible to participate if they (1) were a speech and language therapist who provided input for autistic children or had autistic children on their caseload and (2) worked in the United Kingdom or Republic of Ireland. The web-based cross-sectional questionnaire was disseminated via the first author’s professional social networks, and Clinical Excellence Networks in the SLT field. Participants were encouraged to forward the questionnaire to other eligible colleagues, using a snowball strategy to increase engagement.

Recruitment flyers included information about the research, a QR code, and link to the questionnaire. Upon entering the questionnaire, participants were provided with a participant information sheet, which outlined the rationale for research, research questions, and ethical considerations. Following this, participants were presented with a consent form to participate in the questionnaire and provided informed consent.

### Questionnaire Design and Data Collection

The questionnaire was created using Microsoft Forms to ensure response anonymity was upheld and was open between April 3, 2023 and June 30, 2023. The questionnaire consisted of 23 questions overall, including Likert scales, open-ended responses, and single and multiple-choice questions. Questions included 6 questions on eligibility and basic demographics, 4 on knowledge about VR, 3 regarding previous use of VR, 6 on attitudes toward VR, and 4 on future support needs.

Knowledge about VR involved a self-rating scale about knowledge of VR, multiple-choice questions about different VR technologies, which VR “brands” speech and language therapists had heard of and integration of VR into practice, This question was adapted from Khukalenko et al [23]. Questions regarding attitudes toward VR used a 7-point bipolar Likert scale, to explore SLT reasons for use or nonuse of VR. Facilitators and barriers to adoption of technology were adapted from Albudoor and Peña’s study [24], and conclusions from Brassel et al [20]. Questions on future support needs are directly based on Brassel et al [20], where supporting VR into clinical practice emerged directly as a theme. Open ended questions allow speech and language therapists to consider what they need to support VR into practice, if they would benefit from training and ideas for future research. The questionnaire used display logic to direct participants to relevant questions based on whether they had used VR in clinical practice. Consequently, different numbers of participants answered questions. The questionnaire was not piloted before going live on the web but was deemed coherent by the first author and supervisor.

### Data Analysis

Following questionnaire recruitment, data was exported into Microsoft Excel for analysis. This enabled descriptive analysis of quantitative data, through percentages and counts and was presented visually. Free-text questions were analyzed thematically by the first author, following an inductive thematic analysis approach [25]. This identifed key themes and facilitated a deeper immersion with speech and language therapists’ personal experiences, enabling better understanding and interpretation. Identified themes were discussed and agreed with the supervisor. Following analysis, themes were compared with quantitative data to develop an overall understanding of results, and direct quotes from text were used to contextualize results, increasing confirmability of interpretation.

### Ethical Considerations

This study received ethical approval from the School of Social Sciences, Education, and Social Work Ethics Committee at Queen’s University Belfast (073_2223). All participants provided informed consent and participation was anonymous. No compensation was provided for participation.

## Results

### Overview

Overall, 60 participants gave consent to participate in the questionnaire; however, 7 were not eligible to participate and their data were excluded. Reasons for exclusion include not working within the United Kingdom or Republic of Ireland (n=3), not working with autistic children (n=3), or not completing more than half of the questionnaire (n=1).The low number of responses may reflect the limited use of VR clinically. Results will refer to those who have and have not used VR with autistic children.

### Participant Characteristics

Of the 53 participants who met the eligibility criteria, 48 responses were from women, 4 were from men, and 1 was from a participant who preferred not to comment on their gender. These figures are representative of sex-based employment data which suggests 97% (n=51) of the speech and language therapy profession are female, and only 3% (n=3) are male [26]. Further data relating to age, sector and location are presented in Table 1.

**Table 1.** Showing participant demographics factors.

Characteristic		Number of participants (N=53), n (%)
**Age (years)**		
21-24		11 (21)
25-34		14 (26)
34-45		13 (25)
45-54		9 (17)
55-64		6 (11)
**Sector**		
Public		31 (58.5)
Private		22 (41.5)
**Location**		
England		22 (41.5)
Northern Ireland		14 (26.4)
Scotland		7 (13.2)
Republic of Ireland		6 (11.3)
Wales		4 (7.5)

### Previous Use of VR

In the questionnaire, 3 questions pertained to previous VR use for SLT for autistic children. From 53 respondents, 49 had not used VR and 4 had. From the 4 VR users, 1 had used VR with an autistic child in a social skills context. In total, 3 had not used it in the context of autism but had used it to motivate children for therapy, to trial a VR Lidcombe program, and with stroke users. The Lidcombe Program refers to an SLT intervention used with dysfluency and stammering. Motivation included using gamified elements, and EVA Park had been used with people with aphasia.

### Knowledge About VR

**Figure 1.**
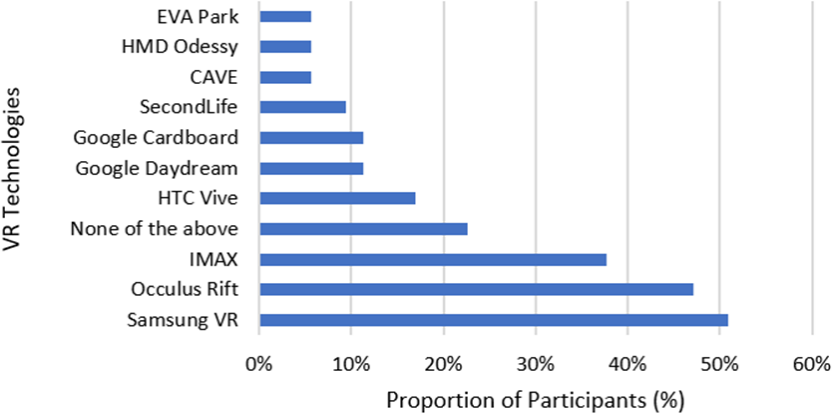
Showing recognition of virtual reality technologies by participants. VR: virtual reality.

Speech and language therapists rated their knowledge on a 5-point Likert scale as excellent, good, average, poor, or very poor. Overall, they demonstrated a mixed self-reported knowledge of VR, with the majority of speech and language therapists who had not used VR, reporting poor (n=22, 41%) and average (n=20, 39%) knowledge of VR. Furthermore, a speech and language therapist exemplified this by reporting: *“My own knowledge is my main limitation.”* Of speech and language therapists who had used VR (n=4), 25% (n=1) rated their knowledge as good, and 75% average (n=3). A total of 53% (n=27) of speech and language therapists who had not used VR were able to correctly identify that VR encompassed desktop computers, CAVE, and HMDs connected to smartphones or laptops. Furthermore, 70% (n=37) perceived HMDs to best describe VR. VR technology brands Samsung VR and Occulus Rift, which use HMDs, were mostly widely recognized, including by speech and language therapists who had used VR. Figure 1 outlines the recognition of various VR. Regarding the integration of VR into clinical practice, 67% (n=35) of speech and language therapists were aware of VR but had not used it in clinical practice. A further 15% (n=7) had become aware of the concept of VR from the survey and had not previously heard of it. VR was more integrated into clinical practice by those had previously used VR, with 50% (2/4) indicating their familiarity and confidence with VR, and 50% (2/4) had fully integrated VR and could creatively apply and use it in clinical practice.

### Attitudes Toward VR

**Figure 2.**
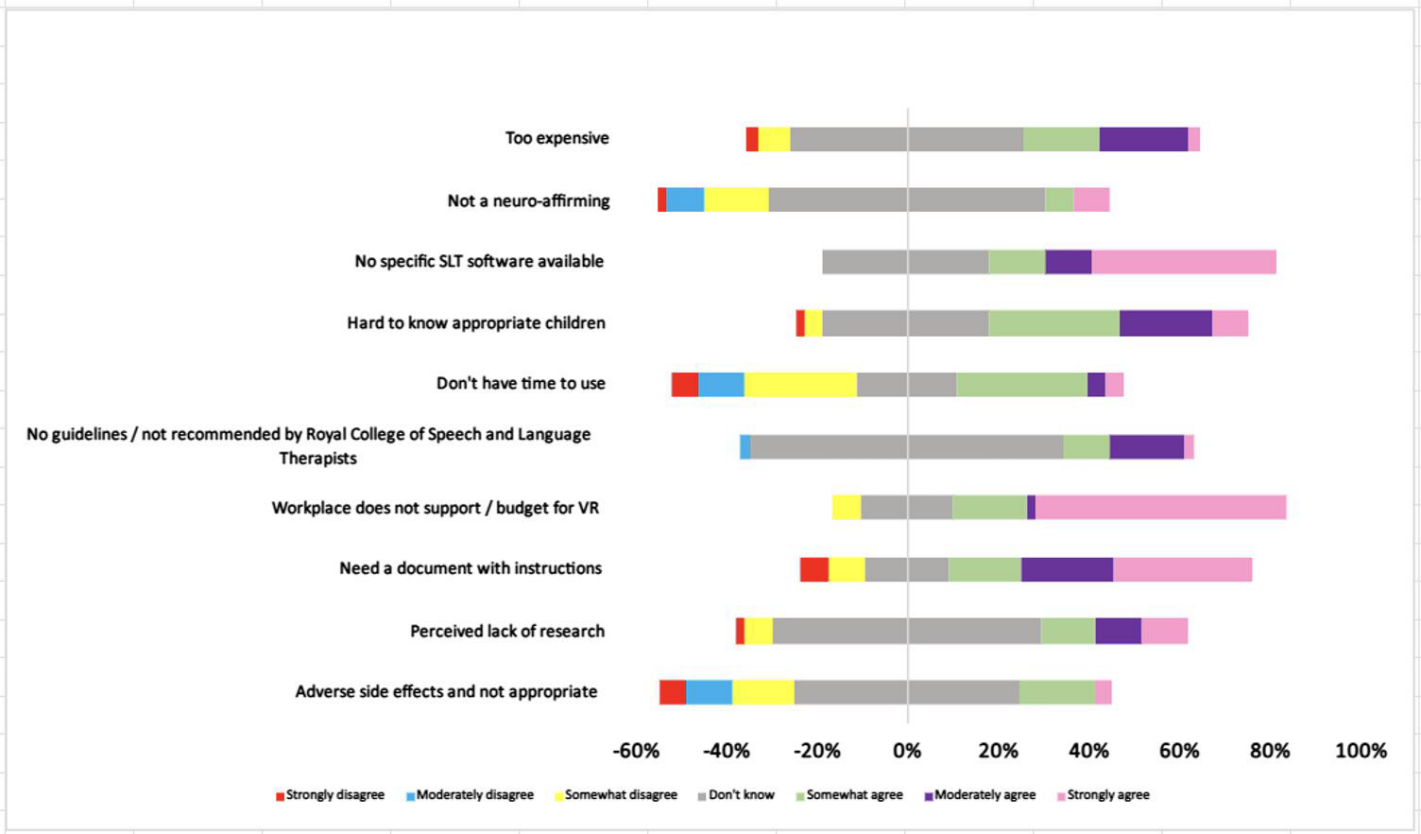
Showing speech and language therapist attitudes toward virtual reality when they had not previously used it in clinical practice. VR: virtual reality.

Participants who had not used VR indicated that workplace budgeting and support, a lack of instruction and guidelines and a lack of specific SLT VR software acted as barriers toward the general adoption of VR into clinical practice (Figure 2).

As illustrated in Table 2, 55% (n=29) of speech and language therapists who had not used VR, strongly indicated that their workplace did not support or budget for VR.

*I’ve never thought of this technology as an option – likely due to budgets and having access to the technology.*

**Table 2.** Showing key quotations from participants that highlight themes and subthemes of future support needs.

Theme and subtheme		Quotation
Training and **education**		*“Knowledge, resource, time to upskill.”*
	Education from specialists	*“Someone to actually visit us in our clinics with the headsets and show us what it is, common misconceptions, how it works, all the different brands and types of VR that are actually available, with all the different features.”* *“Someone who can come in and set up a VR hub and show us what it does, and then what it does for speech therapy”*
	Information of funding	*“There would probably need to be a funding initiative as NHS is a public service and would be unable to just purchase this off the bat.”* *“How to access affordable VR. Is there a way to get funding?”*
	An overview of research	*“Raising awareness of the pros and cons of research”* *“An understanding of how it would support the client group we work with.”* *“Information about case studies/evidence base/research”*
	Practice-based learning opportunities with peer support	*“A new CEN dedicated to the use of VR in practice, where we could talk to SLT’s who are already using it but promote it and can give us tips on what do to next. If they could provide workshops and like online lives or something I would really love that. Like you do it [practice] along with them on a live stream like simulated practice.”* *“Observe other specialist autism SLTs using VR.”* *“Practical based learning, get to use it and experience it, think about creative uses with other SLT’s.”* *“Having someone who is an SLT and knows how to use it teaching you, so they don’t neglect the nuances of the SLT role is important. Otherwise, it might not be neuro affirming for autism.”* *“Training over a few days, interactive get to experience VR, learn to theoretical side and behavioural mechanisms, opportunities to plan for made up clients like you do in university.”*
**Guidelines**		*“Guidelines(!!) this is a really big one I think”*
	VR^a^ user guide and set up	*“A guideline from using it from case history to discharge would be useful for me as an NQP.”* *“I think if a table, with VR types and efficiency for different SLT goals was put into a manual, that would be really helpful for clinical decision making as it would make it easier and way more efficient.”* *“Info on VR implementation: set up, how to support autistic children and parents.”* *“What is it, what therapy looks like, who is it for, how to set goals and measure outcomes.”* *“How to identify who it would benefit, knowing when to use it.”* *“I think an overview about VR and its uses in SLT would be super useful. eg What types of VR there are, and how you could use different software for different applications within SLT - Ax & therapy.”*
	Example therapy ideas	*“Ideas for interventions.”* *“Sample sessions/activities, how to introduce, how to include interests, what to consider from a sensory perspective.”*
	Frequently asked questions	*“Troubleshooting if technical issues arise.”* *“What to do when shit hits the fan and it all goes wrong aswell…”* *“It needs to include 'what ifs' to help troubleshooting with autistic children, as they are all likely to react so differently.”* *“Maybe how to manage side effects because I would panic and so would most of the kids.”*
**Future Research**		*“Realise SLTs in NAH are still in battle mode post covid with severe shortages and whilst most SLTs are interested and can see potential for a range of applications, there are other interventions they are looking into and they have to prioritise. It may be the additional technology bugs/issues/learning curves slide VR into 2nd or 3rd place because we can’t do it all.”*
	Neuro-affirmative uses of VR	*“Ensuring that the virtual reality approach to working with autistic children is neurodiversity affirmative (ie, it isn’t taking a social skills approach and teaching them how to mask). The benefits to autistic children themselves should be outlined and not only to neurotypical adults in their environments.”* *“It would be good to see if VR could be used to explain the autistic child’s perspective to others though, perhaps parents and teachers. Training up the people who work with autistic children and using it as part of advocacy as to how it feels to have social communication difficulties. I saw something similar on Chris Packhams documentary about autism. Although it was done via film, it gave first person POV of sensory differences experienced by an autistic young person.”*
	Effectiveness of a specific SLT^b^ VR tool	“*Parental and SLT views on effectiveness and a range of outcome measures to assess how effective it has been.*” *“Increase the evidence base for different types of VR in autism, and for different goals.”* *“Outcome measures - depending on target of therapy, does VR help achieve these goals or hinder them?”* *“Need to examine generalisation from the VR - learned experience, to the real life experience.”*
	Involving autistic children and families in the design of VR tools	*“Incorporate the ideas and needs of the individual from their own perspective. Client led - developed by clients.”* *“It also might be an idea to actually ask autistic children how they would like to use it when they come to therapy. The best way to understand autism is to ask and include people who have it, especially in any future design or applications.”* *“I think parental views? Do parents think it’s an acceptable therapy when they’ve waited a long time. Does VR provide a new way to work on skills that are a priority for autistic clients and SLT’s - comparing it to approaches being used at the minute.”*
	Consideration of individual needs	“*I would like to see if it impacts sensory differences because I find when the child used it in my clinic he got really over stimulated and had more behavior problems than he did before using it.*” *“It should identify children who may not benefit from it ,ie, who does it work best for.*” *“If there are VR worlds used for autism, I’d also like to know how to keep my young people safe, because I would be worried that their social skills may put them at risk of victimization.*”

a VR: virtual reality.

b SLT: speech and language therapy.

However, 43% (22) of speech and language therapists were not sure if VR was too expensive to purchase. Overall, speech and language therapists knew that their workplace did not budget for or support VR but were unsure about the cost of VR. Most of the speech and language therapists who had used VR indicated that their workplace did support or budget for VR.

In addition, 34% (n=18) of speech and language therapists who had not used VR, did not know if they needed guidelines or a document with instructions, whilst 30% (n=15) strongly agreed that they required guidelines. Overall, most speech and language therapists either wanted guidelines or were not sure. Speech and language therapists who had used VR (n=4) had mixed views. Some did not need instructions or guidelines (2/4, 50%) and some did (n=1 somewhat agree and n=1 strongly agreed). Responses to “no specific SLT software available,” suggested 41% (n=21) of speech and language therapists who had not used VR, strongly agreed that there was no SLT specific software for VR. A further 37% (n=19) remained unsure. This is illustrated in Table 2. Half of speech and language therapists who had used VR did not know (2/4, 50%) if there was or was not SLT specific VR software. Others agreed (n=13, 25%) and disagreed (n=13, 25%).

Attitudes toward using VR specifically with autistic children indicated that speech and language therapists who had not used VR, were unsure about the evidence base (n=29, 55%) and adverse side effects (over 50% [n=26]).

*I don’t know enough about the applications of it within SLT,* ie, *in Assessment & therapy. I would need to know this and have a strong enough evidence base before I could even approach my NHS employers*.

*I would be feared to implement VR, as it may encourage some of the children in my care to disassociate from the world even further*.

In total, 64% (n=33) of speech and language therapists were unsure about the suitability and screening for use of VR with autistic children as a barrier, alongside uncertainties about the neuro-affirming status of VR (n=32, 62%) :

*I cannot see any benefits from using it with autistic children on the grounds of teaching social skills or attempting sensory desensitisation which is harmful, and does not work!!*

Notably, VR was considered to be neuro-affirming by over 30% (n=15) of speech and language therapists who had not used VR. However, 14% (n=7) felt it was not neuro-affirming, as it, “*perpetuates harmful neurotypical stereotypes that autistic children need to change.”* Of speech and language therapists who had used VR (N=4), 25% (n=1) strongly disagreed that VR could have adverse side effects, 50% (n=2) somewhat disagreed, however 25% (n=1) remained unsure. Furthermore, 50% (2/4) of speech and language therapists who previously used VR , felt VR was neuro-affirming and 50% (2/4) did not know.

Around 80% (n=42) of speech and language therapists were willing to use VR in the future and 20% (n=10) were not. Reasons for future usage included: (1) innovation in practice:

*Always willing to explore new ways of working*

and

*VR is in the media ALOT at the minute with the new Apple VR headset, so I think it’s important that practice reality follows these new developments and isn’t stuck in the past*;

(2) the motivational nature of technology:

*It is visual and exciting, I can see the potential for use of VR with this client group*

and

*Because lots of my clients love to use computers/ipads,*

and (3) potential therapeutic impact:

*Another clinical tool to add to successful therapy outcomes,*

*I think that it would support practicing areas such as self-advocacy and perspective taking in a more dynamic way*

and

*If it could be used to improve social communication and language outcomes I would be willing to try it*.

Speech and language therapists who were not willing to use VR gave various reasons including (1) VR may not support neuro-affirming principles,

*I would be worried about social scenarios created by neurotypical people to intervene in helping autistic people practice skills that don’t come instinctively*;

(2) Concerns about sensory issues, and (3) ethical concerns and dissociations,

*Virtual reality represents a disconnection from the real social interactions it [sets] out to improve.*

### Support Needs

The 49 speech and language therapists who had not used VR were asked what they needed to support future research. Key themes emerged including an increased evidence base, workplace support, and training and education. Many speech and language therapists did not answer in full sentences, and this reflects the fragmented quotations used.

An improved evidence base for VR as an SLT tool for autistic children was required and “*having the knowledge to make an informed decision*,” was a priority for many. An improved evidence base was also required to provide a more solid foundation for business cases for employers:

*I don’t know enough about the applications of it within SLT,* ie*, in Assessment & therapy. I would need to know this and have a strong enough evidence base before I could even approach my NHS employers to try and build a business case to support them purchasing it.*

Furthermore, speech and language therapists suggested future research should demonstrate clear quantitative evidence showing the effectiveness and efficacy of VR, clear, replicable outcomes measures for communication and generalization domains, and comparison of VR with standard SLT therapy.

Training and education were also identified as a theme across the data. In total, 83% (n=43) of speech and language therapists felt they would benefit from training, to include an overview of VR technology and guidelines for use. Speech and language therapists suggested a demonstration day, best described by one participant:

*I personally would need a VR road show for autism where you can see what it can do, get to try it and talk with experts and other SLTs using it.*

Speech and language therapists wanted this to include education from specialists about the VR headsets available and appraisal of these, information on how to apply funding, an overview of research and practice-based learning opportunities, with peer support.

SLTs also reported that they needed a VR manual from VR “*procurement to [therapy] discharge.*” This would include a VR user guide and set up, example therapy ideas, and a frequently asked questions guide for troubleshooting and difficulties. Table 2 illustrates quotations which support the above findings.

Finally, specific areas for future research were also identified. This included neuro-affirmative uses of VR, effectiveness of a specific VR SLT tool and outcome generalization, consideration of individual needs, and involving autistic children and families in the designing VR tools. One speech and language therapist indicated that researchers should consider all future research in the current post–COVID-19 context:

*Realise SLTs in NHS are still in battle mode post covid with severe shortages and whilst most SLTs are interested and can see potential for a range of applications, there are other interventions they are looking into and they have to prioritise. It may be the additional technology bugs/issues/learning curves slide VR into 2nd or 3rd place because we can’t do it all.*

## Discussion

### Principal Findings

The aim of this study was to explore speech and language therapists’ knowledge and attitudes toward the use of VR for autistic children. This questionnaire indicated that some speech and language therapists were willing to use VR, discussing barriers, potential uses, and support needed to facilitate the novel technology into practice. Key themes include considerations for VR use, attitudes toward VR and barriers to use, and supporting VR into clinical practice.

Despite being generally unsure about autism-specific considerations, most speech and language therapists were still willing to use VR as an intervention. This may be because some clinicians demonstrate an openness to innovation in practice, with some indicating that they liked to embrace new approaches and keep abreast of developments. Some advantages of VR were highlighted, including its motivational nature for autistic children and access to a range of communication environments, which are also highlighted in literature [11,12]. Despite this, speech and language therapists did not identify many of the other advantages of VR that are reported in literature, such as opportunities for repeated practice of increasing intensity in naturalistic environments, maximizing strong visual skills, or reduced negative social consequences. This indicates that SLTs may be willing to make a leap into VR adoption, relying on their own clinical judgment to evaluate how useful VR is in meeting the needs of autistic children. This demonstrates the perceived usefulness of VR is not only related to the evidence base and ability to facilitate adaptions, but also to its clinical relevance, applicability, and feasibility in the context of workplace, clinical, and individual demands [24].

Notably, some speech and language therapists in this study, who held more negative views toward VR, evaluated the technology from the perspective of autistic children themselves, rather than as clinicians. This may indicate that these clinicians were supportive of neuro-affirming practice, however as demographic data was not collected on approaches to practice, the authors cannot say this definitively. Responses indicated that VR was perceived to be a tool that was based on neurotypical standards, and sought to change the skills of autistic children, rather than foster strength-based approaches and promote environmental adaptions advocated for by neuro-affirming practice [9]. This suggests that neuro-affirming perspectives may be integrated into the technology acceptance model and could be a form of subjective norms, influencing perceived usefulness of VR [27]. Neuro-affirming perspectives in technology adoption, have not yet been explored in research, and this offers an avenue for exploration.

Speech and language therapists opposed to using VR as a clinical tool for autistic children also reported concerns about adverse side effects, dissociation, and sensory impacts, similar to Bryant et al [15] and Brassel et al [20]. This suggests that speech and language therapists may be considering VR in the context of specific patient profiles. Given that autism presents uniquely in many cases, it is difficult to determine optimum autism profiles for use. Although previous research has indicated that VR can be used for sensory integration therapy and reduces stereotypical behaviors in autistic children (Marco et al [28]), this deficit-based research casts doubt over both sensory impacts and use of VR for identity affirming approaches. Indeed, autistic differences in sensory regulation may heighten dizziness and cybersickness, and although autistic children reported feeling calm when using HMDs [12], sensory stimulation impacts individuals differently. VR may allow autistic children to gain access to socializing and friendships through VR that are restricted in real life, resulting in excessive usage. This prolonged emersion may exacerbate sensory and well-being risks including dissociation [29] depression and anxiety [30] and victimization, as social communication differences may increase the risk of trolling. Therefore, VR should be supported by therapist input, and future guidelines for duration safety of use.

Education surrounding the neuro-affirming potential of VR in SLT is likely to influence the attitudes of speech and language therapists and future use of VR. Although some speech and language therapists remained skeptical about the ability of VR to support a strength-based approach that affirms autistic identity, literature indicates that VR has the potential to facilitate neuro-affirmative interventions, such as perspective taking, experience of new situations, and therapeutic interventions for anxiety and phobias [11,31]. Autistic service users felt VR could affirm their identity, facilitating educational experiences, and friendships [11,14]. As VR offers a safe place to explore and practice skills without negative consequences [32], future uses of VR may use the technology to teach skills to repair communication breakdowns, and as an advocacy tool to educate neurotypicals on communication differences.

Poor knowledge of VR was cited as a barrier to adoption, which is surprising given the increased public awareness and commercial availability of VR headsets in recent years, where VR has become a mainstream technology [33]. However, it appears that speech and language therapists struggled to extrapolate the VR represented in the media, to clinical practice applications in autism. This is reflected in literature, where clinical knowledge of VR is perceived to be a barrier to VR adoption in traumatic brain injury [19,21] and indicates the gap between knowledge and practical applications must be bridged to support adoption in autism. Despite this, personal factors such as age, gender, years of experience, and perceptions of digital skills may also impact adoption. Future research should build a clearer picture of links between demographic factors and technology adoption in SLT to understand SLT behaviors that should be targeted for change to support adoption.

Negative workplace attitudes were also highlighted as barriers to adoption of VR and similarly, these risks and barriers have also been highlighted by speech and language therapists using VR for adult communication and cognitive rehabilitation [15,19,21]. This also aligns with the technology acceptance model, by demonstrating that facilitating conditions and technological and institutional factors, play a role in technology adoption [15,24,27]. This indicates that more needs to be done to improve the ability of workplaces to support VR innovation and adoption into clinical practice. For example, grant and reward schemes for managers and trusts embedding VR therapy models.

Fostering positive knowledge and attitudes toward VR in SLT for autism, to overcome barriers, should build on suggestions for education, training, and guidelines. Previous literature in traumatic brain injury has used clinical manuals, workshops, IT support, and courses to make VR easier to use and improve practitioner confidence [21]. Although these approaches are deemed appropriate to facilitate the immersive VR use by speech and language therapists [20], the current suggestions for SLT support may improve upon this previous training model. Support for speech and language therapists using VR with autistic children should target awareness of VR technology, education and practice-based learning, and VR trials and support. Education and training strategies should work in tandem with research knowledge to translate the theoretical into practical. Both speech and language therapists who had and had not used VR, advocated for the development of VR guidelines for autistic children. Bauer et al (2023) [34] has provided detailed extended reality guidelines for paediatric autism interventions, suggesting they should be considered as part of an overall intervention program. However, they also recommend that future interventions should focus on mediation, well-being, and sensorially, social, and cognitive training, which it does not appear to be neuro-affirmative. Although these guidelines provide foundational guidance which speech and language therapists may apply to practice with autistic children, recommendations should be critically appraised on a case-by-case basis. VR SLT created guidelines for traumatic brain injury [20] that may be used as a guide to modify the guidelines by Bauer et al [34] in future research. Participatory design methods should be used in additional research [35] incorporating co-design workshops with speech and language therapists, autistic children, and parents to ensure usage recommendations reflect the needs of service users and speech and language therapists, and are underpinned by neuro-affirmative frameworks.

Overall, this research establishes emerging evidence regarding SLT knowledge and attitudes toward VR. This preliminary research is reflective of Bailey et al [16], where similar affordances and drawbacks are discussed regarding VR in SLT for communication disabilities and the technology acceptance model [27]. Knowledge of VR, subjective norms, including clinical judgment or neuro-affirming practice, and facilitating conditions such as workplace support, contributed to speech and language therapists attitudes toward VR as a clinical tool for autistic children and intention to use the technology.

### Limitations and Future Directions

#### Limitations

Demographic variables were not analyzed in relation to adoption in this study. A fuller profile of respondents may have enabled a more nuanced discussion of VR within SLT autism practice and linked this to current practice. Studies seeking to replicate these findings should evaluate roles in autism care and professional practice. Perhaps speech and language therapists who had more clinical experience with autism, and the appropriateness of interventions, were better able to critically evaluate VR, thus reflecting their more critical attitudes toward VR. Many of these responses showed more depth than speech and language therapists who described a willingness to use VR. Future research should seek to explore the impact of previous training, years of clinical experience, location, and reflective practice on adoption of VR. A specific inclusion criterion, targeting speech and language therapists with substantial experience working with autistic children or using VR is required in future research. Authors should ensure inclusion criteria targets those aged more than 21 years as trained speech and language therapists.

In addition, the questionnaire was completed via web and self-selective, meaning respondents with adequate digital skills responded to the survey. Respondents may have an increased interest in or experience with VR, resulting in a self-reported bias. It may have been beneficial to include an adapted digital skills questionnaire to estimate SLT levels of digital competence, and how this impacted VR adoption. Results would have indicated if competence was related to technology generally, or specifically to VR. Furthermore, a questionnaire limited the depth of understanding of research questions. Focus groups may have enabled a richer exploration of clinician perspectives and future research should seek to use this methodology. Finally, it was difficult to ascertain figures for the number of speech and language therapists working with an autistic caseload in the United Kingdom and Republic of Ireland, from employment figures and therefore no power calculation was carried out. As a result, the authors are unsure if the sample size of 53 accurately sampled this population, and thus if the findings have suitable power. Given the increasing number of autism diagnoses across the United Kingdom, the number of clinicians in these roles may be higher than expected. Therefore, future research should seek to replicate this result with a larger sample size and recruit speech and language therapists from settings where they work predominately with autistic children.

#### Future Research

Patient specific facilitators and barriers to VR SLT interventions should be identified (eg, sensory differences, epilepsy, victimization, dissociation, intellectual disability, and gender) and external features of VR that contribute to effective SLT interventions. Given the link between epilepsy and autism, and the lack of evidence regarding seizure risk with HMDs, side effects are a pertinent area for exploration. Although the risks of HMDs are reportedly low for autistic children [12], it is necessary to establish safety protocols, such as maximum exposure time and treatment intensity required for generalization, to ensure tolerance and comfort. Limiting exposure times may also help reduce the possibility of autistic children dissociating from reality and real social interactions [15].

The neuro-affirming stance of VR was a key issue in this research. Future research should ensure VR research in autism is rooted in strength-based philosophies, given much of the current evidence base for autistic children prioritizes technology-based interventions for social skills and social cognition training [34]. This paper reiterates the need to think beyond a social skills approach, considering other linguistic, educational, and pragmatic domains within the SLT remit. This reflects the systemic change to autism management observed in the SLT literature and current practice standards [8]. However, the authors acknowledge that populations such as developmental language disorder may benefit from VR interventions designed to improve social or communication skills. Furthermore, it may be that a priority setting exercise for neuro-affirmative speech and language therapists would uncover pressing areas for research, which could guide future neuro-affirmative VR research.

SLT-specific software for autism should be designed using participatory research approaches [35] to ensure VR applications are neuro-affirmative, rooted in autistic and family priorities, and contribute to evidence-based practice in socially and ecologically valid ways [36]. This will solidify the use of VR in the care of autistic children. Frameworks such as Design Thinking [37] may facilitate future research allowing this topic to become more defined and to create new ideas to solve barriers to implementation in practice. Feasibility of VR as a clinical SLT tool may follow [38]. Additional research should compare outcomes from VR and traditional SLT clinical tools, enabling a cost benefit analysis for the benefit of clinical decision-making. Suggestions for development include VR applications that facilitate perspective changing and VR as an educational tool.

Finally, an education and training program may be developed for speech and language therapists, regarding VR as a clinical tool for use with autistic children. Key themes are outlined in Multimedia Appendix 1. Future research should explore how a SLT-specific VR training package impacts knowledge and attitudes toward VR as a clinical tool for autism and impacts adoption of VR into clinical practice. First-hand experience of and engagement with VR, may allow speech and language therapists to consider how it may be used with autistic children and for SLT goals. This may also inform future research questions regarding implementation of dosage, minimum and maximum immersion time, and protocols for use, which contribute toward evidence-based guidelines for VR.

### Conclusion

This research demonstrates mixed attitudes toward VR as a tool for autistic children among speech and language therapists. Barriers need to be carefully considered before VR can fully integrate into the SLT clinical toolkit, and this paper makes the following recommendations: (1) future research into VR in SLT should ensure a neuro-affirming approach, including autistic children in development of new software; (2) a stronger neuro-affirming evidence base regarding the use of VR for autistic children in an SLT setting, to contribute to evidence-based practice and enable access to resources; (3) development of a VR education and training program for speech and language therapists to include neuro-affirming practice; and (4) Clinical Excellence Networks should be set up to facilitate peer support among clinicians who adopt VR. From this, working groups of “VR champions” should be formed to provide advice regarding funding procurement and to engage managerial staff using success stories and outcome driven data.

## Supplementary material

10.2196/63235 Multimedia Appendix 1 The following provides a brief outline of suggested themes for an autism specific SLT VR education and training programme.

## Abbreviations

CAVE: Computer Automated Virtual Environment
CHERRIES: Checklist for Reporting Results of Internet E-Surveys
HMD: head mounted display
SLT: speech and language therapy
VR: virtual reality
