# Supplementary material for: Speech and Language Therapists’ Perspectives of Virtual Reality as a Clinical Tool for Autism: Cross-Sectional Survey
Source: JMIR Rehabil Assist Technol. 2025 Feb 27;12:e63235. doi: 10.2196/63235 (PMC11884707; doi:10.2196/63235)
Supplement: Multimedia Appendix 1 [file rehab-v12-e63235-s001.docx]

**Education and training programme**

| **Perceived Barrier** | **Training to address barriers** |
| --- | --- |
| **Awareness of VR technology** | - Input from VR specialist about different types of VR available: CAVE, HMD, different brands - Advantages and disadvantages of different VR types - IT support on to set up VR, training on how to use software’s, and troubleshooting |
| **Education and Practice Based Learning** | Education   - Introduction to neuro-affirming practice, and the evidence base - Overview of current evidence base for clinical application of VR for autism in an SLT setting - Discussions regarding clinical decision making and implications from research - Information about acquiring funding – possible AAC specialist? - Presentation on available VR SLT specific software - Practice Based Learning - Presentations for SLTs who use VR in autism, and older autistic children regarding experiences of VR – screening for VR use; how it can be adapted to meet needs; targeting and setting neuro-affirming goals; session plans for therapy - Considerations for using VR with autistic children – sensory needs, ethics and privacy, disassociation, how to overcome barriers and current draft guidelines for implementation - Integrated case studies – from initial assessment to discharge to facilitate discussions around rationale and clinical decision making |
| **Trial** | - Opportunity for immersion in SLT VR world e.g. using withVR - Discussion and focus group with other SLTs to discuss implications for practice |
| **Continual Support** | - Creation of a clinical excellence network to facilitate continual peer support for VR adoption - Working groups of "VR champions” to engage services and managers – outlining workplace, organisational and clinical governance support requirements |
